# Saliva and tooth biofilm bacterial microbiota in adolescents in a low caries community

**DOI:** 10.1038/s41598-017-06221-z

**Published:** 2017-07-19

**Authors:** Linda Eriksson, Pernilla Lif Holgerson, Ingegerd Johansson

**Affiliations:** 1 Department of Odontology, section of Cariology, Umeå University, Umeå, Sweden; 2 Department of Odontology,section of Pedodontics, Umeå University, Umeå, Sweden

## Abstract

The oral cavity harbours a complex microbiome that is linked to dental diseases and serves as a route to other parts of the body. Here, the aims were to characterize the oral microbiota by deep sequencing in a low-caries population with regular dental care since childhood and search for association with caries prevalence and incidence. Saliva and tooth biofilm from 17-year-olds and mock bacteria communities were analysed using 16S rDNA Illumina MiSeq (v3-v4) and PacBio SMRT (v1-v8) sequencing including validity and reliability estimates. Caries was scored at 17 and 19 years of age. Both sequencing platforms revealed that *Firmicutes* dominated in the saliva, whereas *Firmicutes* and *Actinobacteria* abundances were similar in tooth biofilm. Saliva microbiota discriminated caries-affected from caries-free adolescents, with enumeration of *Scardovia wiggsiae*, *Streptococcus mutans*, *Bifidobacterium longum*, *Leptotrichia sp*. HOT498, and *Selenomonas spp*. in caries-affected participants. Adolescents with *B*. *longum* in saliva had significantly higher 2-year caries increment. PacBio SMRT revealed *Corynebacterium matruchotii* as the most prevalent species in tooth biofilm. In conclusion, both sequencing methods were reliable and valid for oral samples, and saliva microbiota was associated with cross-sectional caries prevalence, especially *S. wiggsiae, S. mutans*, and *B. longum*; the latter also with the 2-year caries incidence.

## Introduction

The most prevalent oral diseases, dental caries and periodontal diseases, are complex infectious diseases where life style and host factors, including behavioural, socioeconomic factors, medical status and genetics, interact^1, 2^. The oral cavity is reported to harbour more than 700 species/phylotypes, among which some species have been cultured^3, 4^. The bacteria are organized in site-specific biofilm communities^5^, with dysbiosis in the tooth surface or gingival pocket communities preceding caries and periodontal diseases^6^.

For dental caries, which result from the demineralization of tooth tissues by acids produced by bacterial fermentation of dietary carbohydrates, the aciduric and acidophilic mutans streptococci (*Streptococcus mutans* and *Streptococcus sobrinus*) have been specifically linked to disease development^6^. However, any acidogenic species, including the mutans streptococci, aciduric non-mutans streptococci, *Bifidobacterium*, *Lactobacillus, Actinomyces*, and *Scardovia* ^7–10^, may contribute to disease development. The relative impact of a species from these genera may vary between populations and within a population over time. For example, it was recently reported that in Romanian adolescents, in whom dental care is limited and disease activity is high, the frequency of both *S. mutans* and *S. sobrinus* was very high compared to in Swedish adolescents, who are exposed to life-long disease prevention and treatment programmes^11^. In 1973, *S. mutans* infection was as prevalent among Swedish adolescents (96%)^12^ as that reported for Romanian adolescents in 2013^11^. Eleven years later (1984), the prevalence had decreased to 77% in Swedish adolescents^12^, and it had decreased to 50% in 2013^11^.

Non-targeted methods are warranted to characterize a disease-associated bacterial community, i.e., methods not limited by present knowledge or expectations. Over recent decades, non-targeted multiplex DNA sequencing of the 16S rRNA gene and taxonomic determination from gene databases have commonly been used^13^. To date, most studies searching for caries-associated microbial patterns have been conducted in young children with severe caries. Only a limited number of studies have targeted adolescents or adults^14, 15^. Currently, Illumina technology is most commonly used for the microbiome characterization of clinical samples. This platform generates many sequences, but taxonomic resolution is limited. Recently, multiplex amplicon sequencing was launched for the Single-Molecule Real-Time (PacBio SMRT, Pacific Biosciences of California, USA) platform, which produces long reads, i.e., up to nine variable regions of the 16S rDNA, and improved taxonomic resolution^16^.

The above described results indicate systematic differences in the oral microbiota between populations by socio-economic status and that shifts occur over time not only at the individual but also at the population level. Therefore, studies targeting various well-characterized populations are required to understand determinants of health and disease under given conditions. The aims of the present study were to (i) characterize and compare saliva and tooth biofilm microbiota via deep sequencing using the Illumina MiSeq and the PacBio SMRT platforms and the Human Oral Microbiome Database (HOMD, www.HOMD.org) for taxa resolution^17^ in a population with long-term caries prevention and low disease activity, (ii) compare saliva and tooth biofilm microbiota in caries-affected and caries-free adolescents cross-sectionally and longitudinally, and (iii) evaluate the relative validity between and within the two sequencing methods. The study will add knowledge concerning saliva and tooth biofilm microbiota in adolescents, representing the most typical situation in Western countries^18^. The study will also be the first to employ the PacBio SMRT amplicon technique for determination of oral microbiota.

## Results

### Illumina MiSeq global sequencing results

Summary data from saliva (n = 64) and tooth biofilm (n = 49) sequencing using the Illumina MiSeq platform are presented in Table 1. The average read length of these sequences was 427 bp (v3-v4), and the average number of cleaned sequences per sample was 71,047 for saliva and 20,056 for tooth biofilm samples. Taxa were found in 10 phyla in both sample types and in 98 and 86 genera in saliva and tooth biofilm, respectively (Table 1). ProbeSeq (http://homings.forsyth.org/index2.html) and QIIME^19^ processing of raw sequences produced similar results (Table 1). In the following sections, data are presented for ProbeSeq processing unless stated otherwise.

**Table 1:** Overview of Illumina MiSeq sequencing by bioinformatic processing and PacBio SMRT sequencing.

	Illumina Miseq		PacBio SMRT
	saliva	tooth biofilm	tooth biofilm
Sample number	64	49	42
Mean sequence length (bp)	429	424	1,360
**ProbeSeq/SMRT specific pipeline**			
Total number of reads	7,858,135	1,328,722	158,705
Quality filtered reads	4,547,033	982,980	142,912
Reads per sample, mean (min-max)	71,047 (46,109–156,263)	20,056 (8,242–34,339)	3,367 (667–8,162)
Phyla^1^	10	10	10
Genera^1^	98	86	77
Species/phylotypes/Genus probes^1^	395	373	345
**QIIME**			
Total number of reads after filtering^2^	8,245,243	1,582,139	—
Quality filtered reads	5,501,208	1,299,986	—
Reads per sample, mean (min-max)	85,952 (60,035–180,901)	26,526 (12,157–49,024)	—
Phyla^1^	10	10	—
Genera^1^	97	80	—
Species/phylotypes^1^	405	368	—

^1^For ProbeSeq and QIIME, OTUs with <10 sequences per OUT were excluded and for PacBio SMRT those with <2 sequences per OUT. ^2^After split library using default QIIME parameters.

### Determination of phyla in saliva and tooth biofilm samples using Illumina MiSeq

*Firmicutes* dominated in saliva (48% abundance, % of all sequences) followed by *Actinobacteria* (20%), whereas the abundances of *Firmicutes* and *Actinobacteria* were more similar (32% and 24%, respectively) in tooth biofilms. *Bacteroidetes, Fusobacteria*, and *Proteobacteria*, which were also represented in all adolescents and both sample types, together constituted 32% and 41% in saliva and tooth biofilm, respectively (Supplementary Tables S1 and S2 **)**.

### Determination of genera in saliva and tooth biofilm using Illumina MiSeq

**Figure 1:**
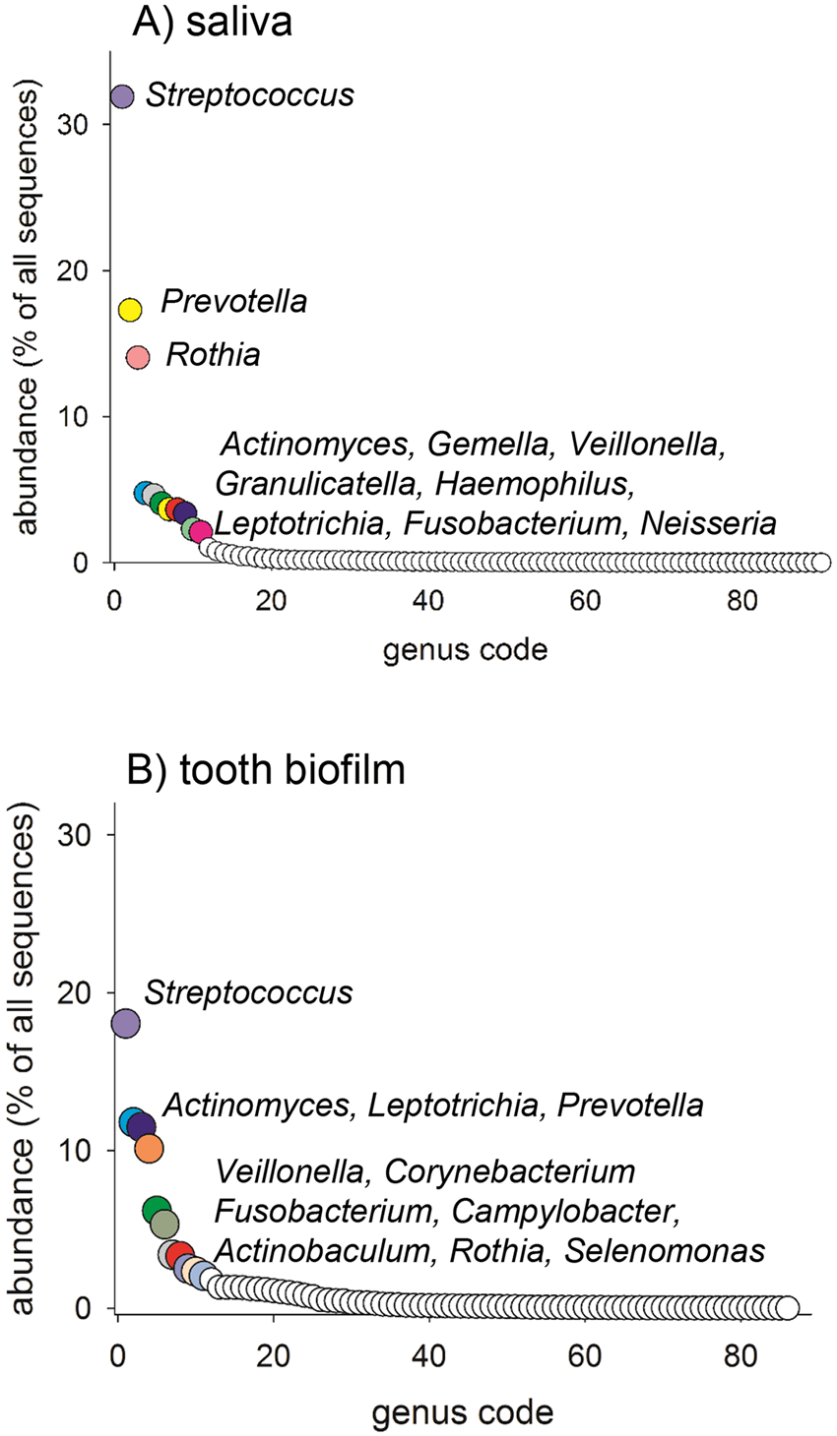
Abundance plot of genera in (**A**) saliva and (**B**) tooth biofilm.

**Figure 2:**
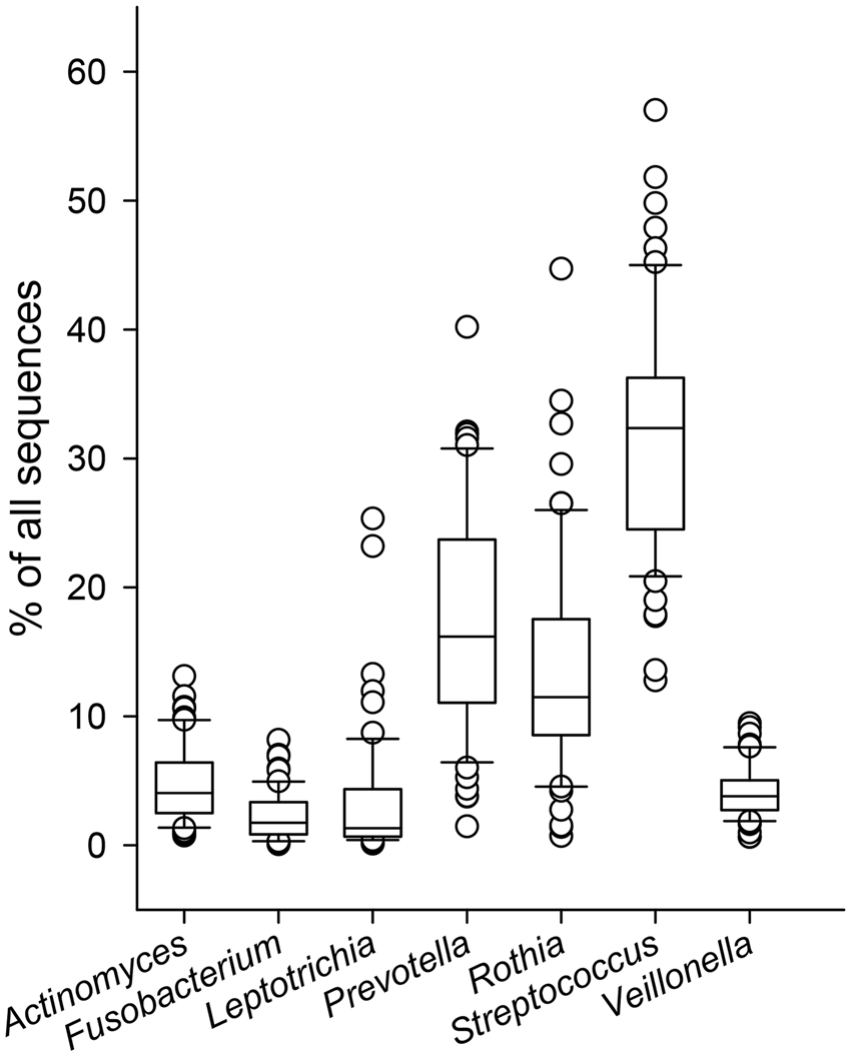
Box plot of genera present in both saliva and tooth biofilm and in all participants. Data are % of all sequences (abundance) in saliva. The bottom and top of the box indicate the first and third quartiles, the line inside the box the median, and the ends of the whiskers the 10th and the 90^th^ percentile values. Outliers are plotted as individual circles.

In total, 11 genera in saliva and 11 genera in tooth biofilm each represented 2% or more of all sequences (Fig. 1A,B, Supplementary Tables S1 and S2). Of these, 7 genera were detected in both sample types (i.e., *Actinomyces, Fusobacterium, Leptotrichia, Prevotella, Rothia, Streptococcus*, and *Veillonella*) (Fig. 1A,B). Although these genera were represented in all adolescents, the individual abundance was highly varied, as exemplified for saliva in Fig. 2. In total, 16 genera were found in both sample types and all adolescents.

At the genus level, *Streptococcus* dominated (32% abundance) in saliva, followed by *Prevotella* (17%), *Rothia* (14%), and 8 additional genera (5-2%) (Fig. 1A). In tooth biofilm, *Streptococcus*, *Actinomyces, Leptotrichia*, and *Prevotella* prevalence rates were more similar (18%, 12%, 11%, and 10%, respectively) as well as 7 additional genera (6-2%) (Fig. 1B). The abundances of all identified genera are presented in Supplementary Tables S1 and S2.

### Species/phylotypes/Genus probes in saliva and tooth biofilm using Illumina MiSeq

In saliva, 30% of all sequences were recognized by the *Streptococcus* Genus probe 4 (recognizing 32 different *Streptococcus* species/phylotypes). Other prevalent species/phylotypes/Genus probes (>2% abundance) in saliva included, in descending order, *Rothia mucilaginosa, Prevotella melaninogenica, Haemophilus parainfluenzae*, species recognized by the *Granulicatella* Genus probe, and *Gemella sanguinis* (Supplementary Table S3). A full list of abundances is shown in Supplementary Table S3, and the species recognized by the Genus probes are listed at http://homings.forsyth.org/Genus%20probe%20list%20for%20website_v2.0.pdf.

In tooth biofilm, 11% of all sequences were recognized by the *Streptococcus* Genus probe 4. Other prevalent species/phylotypes/Genus probes (>2% abundance) in tooth biofilm included *Corynebacterium matruchotii* (4%)*, Actinobaculum sp*. HOT183*, Actinomyces gerencseriae, Actinomyces sp*. HOT448*, Campylobacter gracilis*, species recognized by *Fusobacterium* Genus probe 4*, Leptotrichia wadei, Prevotella melaninogenica, Prevotella nigrescens, Veillonella dispar*, and species recognized by *Veillonella* Genus probe 2 (all between 2 and 3%). A full list of abundances is given in Supplementary Table S4.

#### PacBio SMRT sequencing of tooth biofilm samples

The average read length of the PacBio SMRT sequences was 1,360 bp (v1-v8), and the average number of cleaned sequences per sample was 3,367 (Table 1). Thus, the PacBio SMRT read length was significantly longer, but the sequencing depth was significantly lower than that of Illumina MiSeq sequencing. In total, 345 species/phylotypes in 10 phyla and 77 genera were detected.

Similar to the Illumina MiSeq sequencing of tooth biofilm, the abundances of sequences in the *Firmicutes* and *Actinobacteria* phyla were similar (23 and 26%, respectively), followed by *Bacteroidetes, Fusobacteria* and *Proteobacteria* (in total 39% of all sequences). *Actinomyces* was the most abundant genus (19%), followed by *Streptococcus* (10%), *Prevotella* (9%), *Leptotrichia* (8%), and *Fusobacterium*, *Corynebacterium*, and *Veillonella* (all 6%). The most frequently detected species was *Corynebacterium matruchotii* (5% abundance). The full lists of abundances are given in Supplementary Tables S5 and S6.

#### Core microbiome in saliva and tooth biofilm samples

Using Illumina MiSeq, 50 species/phylotypes/Genus probes were detected in all saliva samples, and 23 were detected in all tooth biofilm samples (100% prevalence). Nineteen of these “core species” were shared by saliva and tooth biofilm (Supplementary Tables S3 and S4). The PacBio SMRT analyses confirmed that *Campylobacter gracilis, Corynebacterium matruchotii, Streptococcus mitis, Veillonella dispar*, and *Veillonella parvula* were present in all of the analysed tooth biofilm samples (Supplementary Table S6).

### Tooth biofilm microbiota in adolescents with or without caries

#### Study group characteristics

The characteristics of the 17-year-old adolescents in the caries-affected versus caries-free group are presented in Table 2. In addition to higher caries scores, the former group had a higher BMI (p = 0.033), more colony forming units (CFUs) of mutans streptococci in saliva (cultivation) (p < 0.001), and a higher *S. mutans* prevalence by PCR (p = 0.001). Additionally, they brushed their teeth less frequently (p = 0.012). Caries, caries-associated variables, and detected taxa were not systematically different between boys and girls.

**Table 2:** Participant characteristics.

	Caries prevalence at baseline			Caries increment		
	Caries free (n = 26)	Caries (n = 37)	P- value	No (n = 38)	Yes (n = 17)	P- value
Boys/Girls, %	46.2/53.6	45.9/54.1	0.987	52.6/47.4	35.3/64.7	0.232
Number of teeth, mean (95% CI)	28.0 (27.6–28.4)	27.6 (27.2–28.0)	0.109	28.1 (27.6–28.5)	27.9 (27.3–28.6)	0.709^4^
Caries status						
DFS, mean (95% CI)	0	5.8 (4.5–7.2)	<0.001	0	2.3 (1.5–3.1)	<0.001^5^
DS, mean (95% CI)	0	1.2 (0.7–1.6)	<0.001	0	0.53 (0.12–0.94)	<0.001^5^
Smoking, %	7.7	2.7	0.360	2.6	17.6	0.048^4^
Swedish snuff, %	0	10.8	0.083	10.5	11.8	0.892^4^
BMI, kg/m^2^, mean (95% CI)	21.4 (20.3–22.4)	23.3 (21.8–24.9)	0.033	22.3 (21.2–23.5)	23.5 (20.5–26.6)	0.331^6^
Saliva flow rate, ml/min, mean (95% CI)	1.3 (1.0–1.6)	1.4 (1.2–1.7)	0.523	1.3 (1.1–1.5)	1.4 (1.0–1.7)	0.682^6^
Tooth brushing, %^1^	92.3	64.9	0.012	78.9	64.7	0.263^6^
Additional fluoride, %^2^	—	—		23.7	58.8	0.011^4^
Sweet snacks/day, mean (95% CI)	2.5 (1.6–3.4)	3.8 (2.2–5.3)	0.186	3.0 (2.1–4.0)	3.1 (1.0–5.3)	0.926^6^
*Mutans streptococci* by culture, CFU/ml^3^	1.3 (0.6–2.0)	3.1 (2.6–3.7)	<0.001	2.4 (1.7–3.1)	2.8 (1.9–3.6)	0.489^6^
*S. mutans* by PCR saliva, %	44.0	83.8	0.001	64.9	82.4	0.191^6^
*S. mutans* by PCR tooth biofilm, %	36.0	64.9	0.025	51.4	64.7	0.359^6^

Participants were 17 years old at recruitment with follow-up 2 years later. Caries was defined as DFS >0 and caries-free as DFS = 0. Caries increment was defined as DFS at follow-up - DFS at baseline. Differences in subject distributions were tested with Chi^2^ test among groups, and differences between group means were tested with Student’s t-test. Adjustment for sex or number of teeth did not alter the relation between groups for any variable. ^1^Proportion brushing twice a day or not are compared. All brushed at least 3–4 times a week. ^2^Additional fluoride refers to rinsing, varnish, tablets, gel treatments. Information only available in 19-year olds. All used fluoridated tooth paste. ^3^Mean (95% CI) for log 10 of colony forming units (CFU) per ml chewing stimulated saliva. ^4^Information at 19-years of age. ^5^2-year caries increment. In adolescent with no 2-year increment, 52.2% were caries-free and 47.4 had caries at baseline, whereas those 94.1% with 2-year increment had caries at baseline. ^6^Data are based on information at 17-years of age.

#### Microbiome profile by caries status

**Figure 3:**
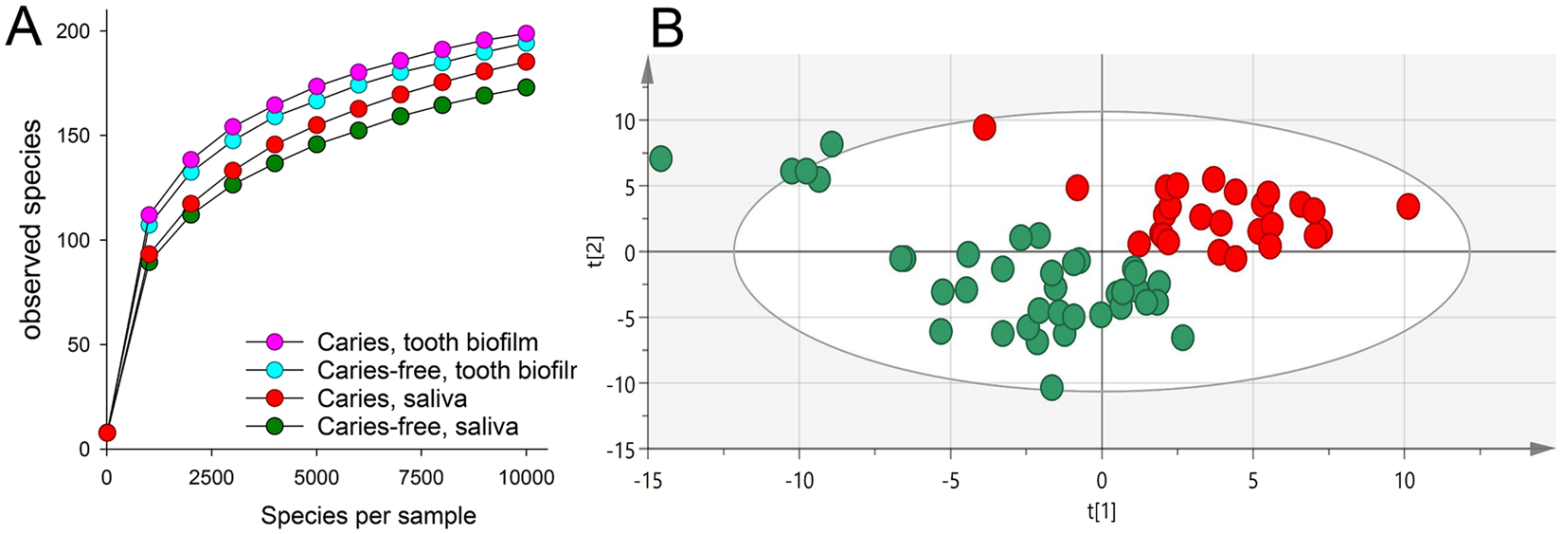
Species richness in saliva and tooth biofilm samples from 17-year old Swedish adolescents and subject clustering. (**A**) Rarefaction curves showing species richness based on number of species by species per sample in saliva and tooth biofilm samples. (**B**) Principal component analysis (PCA) scatter plot separating the caries-affected (red dots) from caries-free adolescents (green dots) based on microbial taxa detection in saliva and lifestyle confounders.

Microbiota richness. The species richness in saliva and tooth biofilm samples did not differ between subjects with or without caries (Fig. 3A).

Microbiota diversity. PCA modelling with species/phylotypes/Genus probes in saliva (Illumina MiSeq ProbeSeq processing), tooth brushing, BMI, sugar intake, and tobacco use included as potential confounders separated caries-affected adolescents from those who were caries free (Fig. 3B). Similarly, PCoA modelling by QIIME identified Operational Taxonomic Units (OTUs) tended to separate most caries affected subjects from caries free subjects (Supplementary Fig. 1).

**Figure 4:**
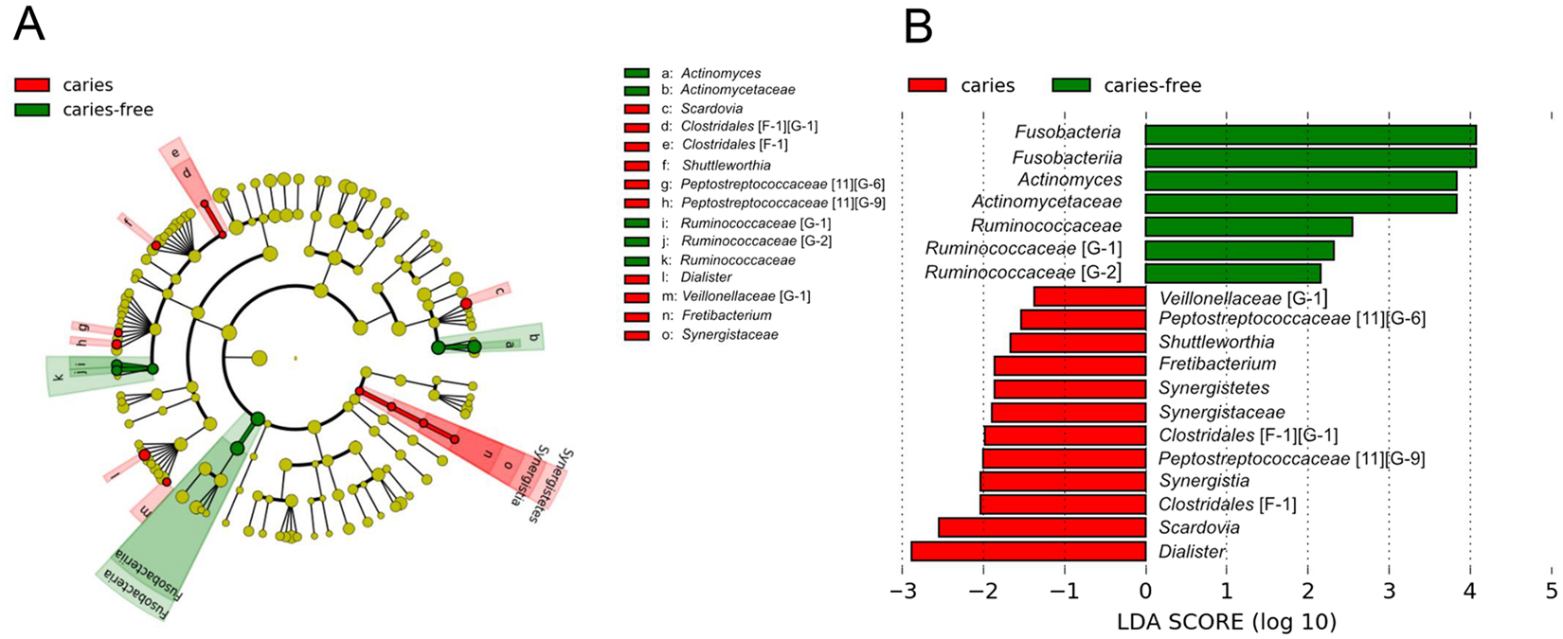
LEfSe analysis of saliva microbiota in caries versus caries-free adolescents. (**A**) Cladogram generated by LEfSe indicating differences at phylum, class, family and genus levels between the two groups. Each successive circle represents a phylogenetic level. Regions in red indicate taxa enriched in caries affected while regions in green indicate taxa enriched in caries-free subjects. Differing taxa are listed on the right side of the cladogram. (**B**) Bar graph showing LDA scores.

A linear discriminant analysis (LDA) effect size-based cladogram, in which successive circles represent a phylogenetic level (phylum, class, family, genus) indicated that species in the *Synergistetes* phylum, *Synergistia* class*, Clostridiales* [F-1] and *Synergistaceae* families, and *Dialister*, *Scardovia, Clostridiales* [F-1][G-1], *Fretibacterium, Shuttleworthia, Peptostreptococcaceae* [11] [G-6] and [11][G-9], and *Veillonellaceae* [G-1] genera were enriched in subjects with caries, whereas taxa in the *Fusobacteria* phylum, *Fusobacteriia class, Actinomycetaceae* and *Ruminococcaceae* families, and *Actinomyces, Ruminococcaceae* [G-1], and [G-2] genera were enriched in caries-free subjects (Fig. 4A). These results are also illustrated in an LDA bar graph (Fig. 4B).

**Figure 5:**
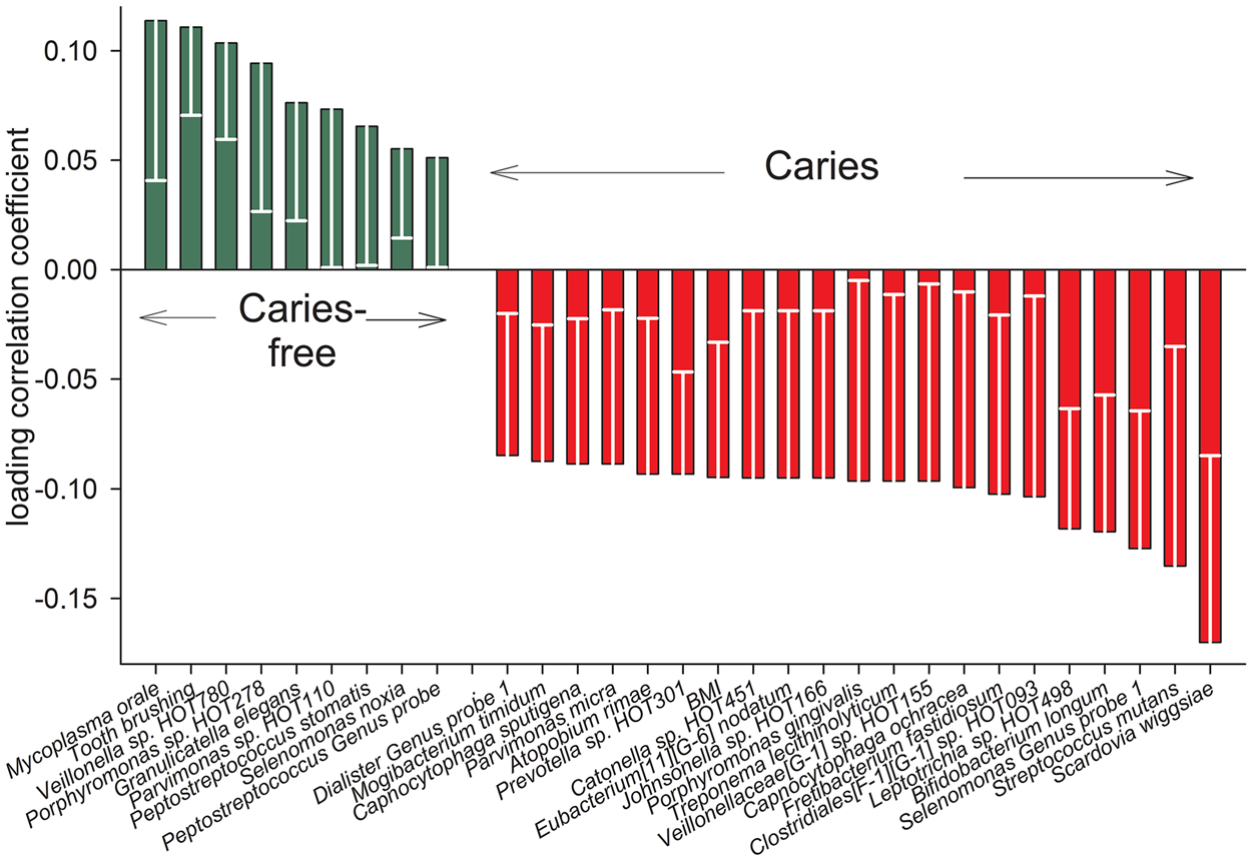
PLS loading column plot. Mean (95% CI) PLS correlation coefficients for the top 20 taxa associated with having caries and all taxa associated with being caries-free. The PLS model included caries as dependent variable and saliva detected species level taxa, sex, BMI, oral hygiene, and sweet snacking, as the independent block. w describes the PLS weights from the combination of the original variables in the X-swarm and c the same for the Y-swarm. A variable is considered statistical significant when the 95% CI of the correlation coefficient does not include zero.

Partial least squares (PLS) modelling with caries status (DFS yes/no) at 17 years of age as the dependent variable and an independent block of species/phylotypes/Genus probes (yes/no) in saliva along with the same set of confounders as in PCA yielded a model with four significant components and a cross-validated predictive capacity (Q^2^) of 37% of the two first components. The strongest associations (PLS correlation coefficients >0.1) with the presence of caries were found for *Scardovia wiggsia*e, *Streptococcus mutans, Selenomonas* Genus probe 1, *Bifidobacterium longum*, and *Leptotrichia sp*. HOT498, whereas *Mycoplasma orale* and *Porphyromonas sp*. HOT278 were most strongly (PLS correlation coefficients >0.1) associated with being caries free. In addition, BMI was significantly associated with having caries, and tooth brushing was significantly associated with being caries free (Fig. 5).

The findings of the multivariate PLS model were consistent with univariate comparisons in which *S. wiggsiae* was found in the saliva of 100% of individuals with caries compared to 65.4% of caries-free adolescents (p < 0.0001, which was significant after false discovery correction) (Supplementary Table S3). Other PLS identified caries-associated species prevalence rates were also higher in univariate comparisons (p ≤ 0.008) but did not reach significance after false discovery correction, i.e., *S. mutans, Selenomonas* Genus probe 1, *B. longum*, and *Leptotrichia sp*. HOT498. In addition, the mean percent of all sequences (abundance) of S. *mutans was* significantly higher (p < 0.0001) in caries-affected than in caries-free adolescents and tended to be higher (p ≤ 0.008) for *Bifidobacterium longum, Fusobacterium nucleatum subsp. nucleatum*, and *Selenomonas* Genus probe 1, whereas the opposite was found for *Mycoplasma orale* and *Porphyromonas sp*. HOT278 (both p = 0.009) (Supplementary Table S3).

For tooth biofilm microbiota (Illumina MiSeq, ProbeSeq processing), PCA did not indicate any clustering of subjects, and PLS modelling was not applied. Similarly, neither the prevalence nor abundance displayed a significant difference between the two caries groups, although the prevalence of the *Capnocytophaga* Genus probe 1 and *Veillonella rogosae* tended to be higher for caries-affected than caries-free adolescents *(*p ≤ 0.008) as did the abundance of *S. mutans* (Supplementary Table 4).

Similarly, PCA modelling of PacBio SMRT sequences did not indicate the clustering of subjects by tooth biofilm taxa, and no significant differences were found in univariate analyses between the two groups. However, caries-diseased adolescents tended to have a higher prevalence of *Actinobaculum sp*. HOT183, *Actinomyces sp*. HOT171*, Granulicatella adiascens*, *Rothia aeria*, and *Veillonellaceae* [G-1] *sp*. HOT150 (p ≤ 0.008). *Actinobaculum sp*. HOT183 and *Veillonellaceae* [G-1] *sp*. HOT150 also tended to be more abundant in caries-affected than caries-free adolescents, whereas *Fusobacterium nucleatum subsp. polymorphum* tended to be more abundant in the caries-free group (p ≤ 0.008) (Supplementary Table 6).

#### Saliva microbiota and the 2-year caries increment from 17 to 19 years of age

PCA did not reveal any systematic clustering of subjects by 2-year caries increment but a higher proportion of adolescents who had developed caries over the 2-year follow-up period were smokers and had received additional fluoride treatments (Table 2). However, when 2-year mean caries increments were compared in adolescents with or without *B. longum* in saliva (means adjusted for sex, oral hygiene, sugar intake, BMI, and tobacco use by general linear modelling (glm)), the former group had a significantly higher increment (2.1 versus 0.5 new caries lesions, p = 0.005). Adolescents with *S. mutans* or *S. wiggsiae* had a numerically higher 2-year mean caries increment (both 0.8 versus 0.3), but the differences did not reach significance (p > 0.05).

### Method validations

#### Reliability of sequencing analysis

**Figure 6:**
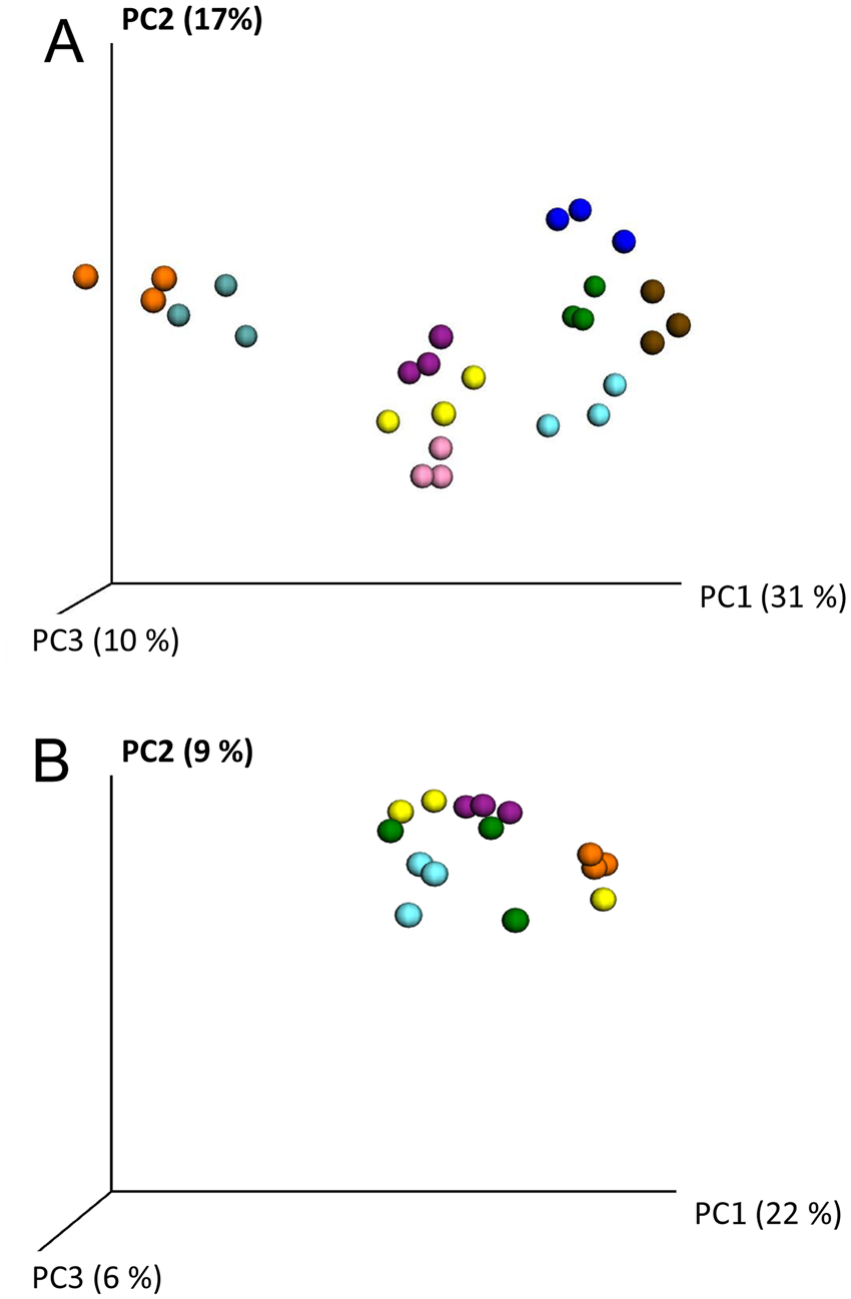
Reproducibility of Illumina Miseq sequencing of saliva samples. PCoA plots of (**A**) same samples run in triplicate, and (**B**) three independent samples collected from five subjects every second day during a week. Same colour indicates same sample or subject.

Nine tooth biofilm DNA samples in triplicate and saliva samples from 5 subjects collected one day apart during one week were analysed using the Illumina MiSeq platform. Triplicate samples clustered close together, and samples were distinctly separated from each other (Fig. 6A). The repeated saliva samples clustered together for three subjects, whereas the sample from one day deviated for one subject and for all three samples in one subject (Fig. 6B).

#### Validity

Illumina MiSeq sequencing detected all 10 species in the *Streptococcus* mock correctly among OTUs with ≥10 sequences but also suggested 5 OTUs as unnamed *Streptococcus* phylotypes. Of the 20 *Lactobacillus* species in that mock, 16 were correctly identified among OTUs with ≥10 sequences, and 4 could not be detected because they were not included in the HOMD database (*L. curvatus, L. colehominis, L. gallinarium*, and *L. graminis*). Similarly, with ≥10 sequences/OTU as the cut-off, 25 (of 32) species from the mixed genera mock were correctly identified. For the 8 non-identified species, 1 was represented by 2 sequences only, and 7 species of different genera were not detected at all. The specificity was low for OTUs with fewer than 10 sequences. The number of sequences for the detected species varied greatly, i.e., 38,862 sequences for *S. mutans* and 3,721 for *S. sanguinis* in the streptococcus mock.

The sensitivity and specificity for *S. mutans* detection by both Illumina MiSeq and PCR were calculated. The sensitivity values for both saliva and tooth biofilm were high (0.98 and 0.90, respectively). ROC curves were calculated (Supplementary Fig. 2), and the area under the curve was 0.94 for saliva and 0.74 for tooth biofilm.

PacBio SMRT sequences of the *Streptococcus* mock identified 9 of 10 species with >1 sequence. The 10^th^ was present with 1 sequence. In the *Lactobacillus* mock, 16 of 20 species were detected, with the same undetected species described above, i.e., 4 species not in the HOMD database. Similarly, a more than 10-fold variation in the number of detected sequences per species was observed, i.e., for streptococci, from 553 for *S. sobrinus* to 49 for *S. sanguinis* (in addition to *S. mitis* with 1 sequence); for lactobacilli, from 343 for *L. paracasei* to 10 for *L. crispatus*.

In addition, a negative control sample (ultra-pure water) subjected to Illumina MiSeq sequencing yielded 620 reads, which was far below the acceptance criterion for this method, i.e., ≥5,000 reads per sample.

#### Correlation between saliva and tooth biofilm taxa

Spearman correlation coefficients between abundances in the saliva and tooth biofilm by Illumina MiSeq sequencing varied from 0.9 to −0.1 for various genera, reflecting the similarities and differences between the hard- and soft-tissue niches. The Spearman correlation coefficients between saliva and tooth biofilm abundances for the caries-associated taxa were as follows: *S. wiggsiae* and *Leptotrichia sp*. HOT498 r = 0.6 (p < 0.001), *S. mutans* r = 0.7 (p < 0.001), and *B. longum* and *Selenomonas* Genus probe 1 r = 0.3 (p < 0.05).

## Discussion

In the present study, saliva and tooth biofilm samples were analysed using Illumina MiSeq, and tooth biofilm samples were also analysed using PacBio SMRT sequencing. This study is the first to report the use of the latter technology on oral samples. PacBio SMRT sequencing confirmed the results from Illumina MiSeq sequencing at the phylum and genus levels and partially at the species level, including the predominance of *C. matruchotii* in tooth biofilms. *C. matruchotii* was overlooked in many previous studies using PCR-based or metagenomic sequencing but was recognized as a prominent species in metatranscriptome^20^, metaproteome^21^, and spectral imaging fluorescence hybridization^5^ analyses. Saliva microbiota, but not tooth biofilm microbiota, distinguished adolescents with caries from caries-free adolescents, with *S. wiggsiae, S. mutans, B. longum, Leptotrichia sp*. HOT498 and species recognized by *Selenomonas* Genus probe 1 (*S. noxia* and *Selenomonas sp*. HOT140) as the most influential taxa for group distinction.

The dominant taxa in saliva and tooth biofilms largely coincided with those reported in previous studies, especially at the phylum and genus levels^14, 20–23^. Thus, according to the Illumina MiSeq results, phylum *Firmicutes* and genus *Streptococcus* dominated in saliva, whereas abundances of the phyla *Firmicutes* and *Actinobacteria* were similar in tooth biofilm samples, and the *Streptococcus* genus was only slightly more abundant than *Actinomyces*. PacBio SMRT sequencing also identified similar levels of *Firmicutes* and *Actinobacteria* but a considerably higher abundance of *Actinomyces* than *Streptococcus*. Generally, studies using PCR-based 454 FLX+ pyrosequencing or Illumina MiSeq sequencing report a higher abundance of *Streptococcus* than *Actinomyces*, but the opposite has been indicated in newer metatranscriptome^20^ and metaproteome^21^ studies. The reason for the discrepancy between the two sequencing methods observed in the present study cannot be determined but likely relates to method-specific biases/strengths and possibly the difference in sequencing depth between the two methods. Five samples were placed per PacBio SMRT cell, but fewer would have been preferential making cost of PacBio SMRT sequencing compared to Illumina MiSeq a hindrance. However, comparisons with other studies must be performed with caution because the sequencing methods, age of the study subjects, dentition (primary versus permanent teeth), and socio-economic conditions of the target populations differed between most studies. Although the PacBio SMRT method has been used for samples from non-oral sites and mock communities^24, 25^, the fact that the present study was the first to report tooth biofilm microbiota limits comparisons with other studies. Thus, in accordance with results from metaproteomic profiling^21^, PacBio SMRT identified *Actinomyces*, *Streptococcus*, *Corynebacterium*, *Leptotrichia*, and *Veillonella* as the five most abundant genera, whereas *Rothia* was less abundant by PacBio SMRT than by both metaproteomic profiling^21^ and Illumina MiSeq sequencing in the present study.

The structure of tooth biofilm has recently been elegantly illustrated by spectral imaging fluorescence *in situ* hybridization^5^. The arrangement was referred to as a “hedgehog structure” with clustering of species in *Corynebacterium, Streptococcus, Porphyromonas, Haemophilus/Aggregatibacter, Neisseriaceae, Fusobacterium, Leptotrichia, Capnocytophaga, and Actinomyces*, with dominance of the 3 first genera and a central role of *C. matruchotii*. In the present study, most of these (*Corynebacterium, Streptococcus, Porphyromonas, Fusobacterium, Leptotrichia, Capnocytophaga, and Actinomyces)* were found in all or nearly all of the adolescents regardless of the sequencing method, and *Neisseria* and *Haemophilus/Aggregatibacter* were found by the more extensive Illumina MiSeq sequencing*. C. matruchotii*, which was also detected in all adolescents by both sequencing methods, was the most abundant species by PacBio SMRT and ranked second by Illumina MiSeq, reflecting its central position in the hedgehog or so-called “corn-cob formation”^5^.

Overall, saliva sequences represented more genera and species than tooth biofilm sequences. This may reflect that saliva is in contact with various surfaces in the mouth, i.e., mucosal surfaces, tongue epithelium, teeth, and gingival margins, rather than transient bacteria or the effect of a higher sequencing depth in the saliva than in tooth biofilm samples. By contrast, bacteria on teeth are selectively recruited by discriminatory attachment to tooth tissue adhering saliva proteins/peptides and interactions with neighbouring bacteria^4, 5, 22, 26^. Among the earliest saliva proteins/peptides that adhere to a clean tooth surface are the acidic proline-rich proteins, statherin and cystatins^27^, which unfold their binding epitopes for *Streptococcus* and *Actinomyces* upon tooth attachment^22^. Yet, other explanations cannot be ruled out, such as an influence of total amount of DNA captured in saliva versus tooth biofilm samples and systematic species differences in DNA retrieval. Notably, a mixture of enzymes were used in the present study to break the resistant cell walls of Gram positive bacteria, and Gram positive species in the mock with mixed genera, i.e. species in *Bifidobacteria, Scardovia* and *Rothia*, were detected in numbers that were similar to Gram negative species.

Some validations were performed in the present study, including negative and positive controls and repeated analyses. It was concluded that false positives due to contamination were negligible, and reproducibility was high for Illumina MiSeq sequencing; however, the sample composition may vary daily in some individuals. The latter is consistent with results reported by David *et al*.^28^. The detection specificity at the species level was acceptable for both the Illumina MiSeq and PacBio SMRT methods when the appropriate cut-offs were applied for taxa allocation. Unexpectedly, the numbers of sequences per species in the mock varied largely, even though equal volumes of bacterial suspensions of the same OD were added; therefore, this finding should be considered in the data interpretation and modelling. We chose to use a more conservative dichotomous variable (yes/no) in statistical models of taxa in the independent data block. The present study population was characterized by a significant caries reduction over the last five decades^29^, regular high-standard dental care, including compulsory preventive strategies, from early childhood^18^, and generally slow development of caries symptoms. The selected conservative approach better addressed time fluctuations in microbiota abundances. Use of the ProbeSeq or QIIME approach for the bioinformatics of Illumina sequences did not confer any substantial difference in the interpretation of results. Because the ProbeSeq approach has the advantage of clustering species with very high 16S rDNA identity into a Genus probe group, this approach was preferred. Thus, for the present clinical research question, we found the ProbeSeq processing of oral samples and dichotomous measures most suitable and that our results supported the previously reported validity of ProbeSeq-processed Illumina MiSeq data^30^.

In the present study group, saliva microbiota separated caries-affected from caries-free subjects, whereas microbiota from tooth biofilm did not separate these groups. There are several plausible explanations for this finding, including that tooth biofilm samples only represent sampled surfaces, whereas saliva is the pool from which tooth colonizers are recruited and reflects most surfaces in the mouth. In contrast to our findings, it has been claimed that saliva does not reflect tooth biofilm microbiota and variations among single tooth surfaces^31^. In the present study, the caries-associated bacterial species in saliva were consistent with those found in previous studies that used various techniques (cultivation, PCR, DNA hybridization chips) on tooth biofilm samples, and the abundances of the caries-associated taxa in saliva were significantly correlated with the corresponding abundance in tooth biofilms. This result supports the use of saliva; however, as recommended^32^, a combination with more traditional techniques, such as culturing and PCR, should be used for optimal identification. Thus, based on the present findings, we suggest that chewing-stimulated saliva is valid for the characterization of caries-associated microbiota in large-scale epidemiological studies, but this finding has been contradicted by others^32, 33^.

The species that were most strongly associated with caries were *S. wiggsiae, S. mutans, B. longum, Leptotrichia sp*. HOT498, and species detected by *Selenomonas* Genus probe 1*. Selenomonas* Genus probe 1 targets *S. noxia* and *Selenomonas sp*. HOT140, of which *S. noxia* has been found to be increased in patients with gingivitis^34^. Thus, a higher prevalence of *Selenomonas* Genus probe 1 likely reflected less frequent tooth brushing and gingivitis in caries-affected adolescents rather than an aetiological role in the caries process. Consistent with the present finding of *Leptotrichia sp*. HOT498 being more prevalent in the saliva of caries-affected than caries-free adolescents, it was recently reported that this species was more prevalent in severely caries-affected Romanian adolescents than in Swedish adolescents with a low caries prevalence^11^. This difference was found in tooth biofilms and for sequences obtained by the LibL 454 FLX+ pyrosequencing option covering the first four variable regions of the 16S rRNA gene. However, *S. wiggsiae, S. mutans*, and *B. longum* are well-documented aciduric and acidophilic species that have been associated with caries in many earlier studies^6–10, 35, 36^. Among these three species, *B. longum* was significantly associated with the 2-year caries incidence; however, because the number of subjects carrying *B. longum* was low, the association must be evaluated in a larger sample. In addition, smoking and having topical fluoride treatment besides a fluoride toothpaste were associated with 2-year caries increment. Smoking is well documented to be associated with caries prevalence and even in this small study sample the association was evident. In contrast, fluoride is a well documented caries protective compound. It may therefore seem contradictory to find such treatment more prevalent among adolescents with disease development. However, in the study population persons with a high risk to develop caries and/or high caries activity have annual dental visits and preventive measures are required. For this reason fluoride treatment in this population reflects caries treatment in high caries subjects and not the effect of fluoride per se.

The strengths of the present study include that (i) the study groups were larger than those of previous studies, and the study extended knowledge beyond early childhood caries and caries in deciduous teeth, (ii) the criteria for sequence inclusion were based on results from simultaneously analysed mock samples, (iii) the sparsely used PacBio SMRT method with improved taxa resolution was applied, (iv) the study population represented populations with a substantial caries decline in the 20^th^ century, and (v) prospective caries incidence was evaluated. The weaknesses include that (i) tooth biofilm could not be analysed in all participants due to limitations in DNA accessibility, (ii) it cannot be assured that every single tooth surface was sampled, (iii) the sequencing depth was low for PacBio SMRT, and (iv) saliva was not analysed using PacBio SMRT.

## Conclusions

Based on the present findings, we conclude that saliva microbiota can separate adolescents with caries from caries-free adolescents in a low-caries population with daily tooth brushing using fluoridated tooth paste^18^. The three most influential species were *S. wiggsiae, S. mutans*, and *B. longum*. We also showed that *C. matruchotii* is enriched in tooth biofilms relative to saliva and that overall PacBio SMRT sequencing covering most of the 16S rDNA supported the findings of Illumina sequencing. However, some discrepancies need to be followed up in future studies.

## Methods

### Study subjects

Seventeen-year-old adolescents, who were caries-free (no present or previous caries) or had present caries activity and a high caries risk^37^, were invited to participate. Subjects were consecutively recruited from three Public Dental Health Care Clinics in the city of Umeå, Sweden. The exclusion criteria were that the adolescent (or caregiver) did not consent, had a chronic disease or was on medication, had taken antibiotics during the latest 6 months, or was unable to communicate in Swedish or English. The caries status was evaluated at 17 and 19 years of age. Baseline data were collected in 2013. Twenty-six caries-free and 37 caries-affected adolescents were recruited, and 55 of these subjects were re-evaluated at 19 years of ages. The population from which the participants were recruited has an overall low caries prevalence, as well as annual dental care, including preventive measures, provided since 2–3 years of age (http://www.socialstyrelsen.se/nationalguidelines).

### Sampling for microbiota analyses

Whole saliva (5 ml) was collected into ice-chilled sterile test tubes while chewing on a 1-g piece of paraffin wax. Next, 100 μl of fresh saliva was transferred to a transport medium for cultivation, the remaining saliva centrifuged at 4 °C (13,000 rpm for 5 min), and the pellets stored at −80 °C until DNA extraction and amplicon sequencing by Illumina MiSeq (http://www.illumina.com).

Supragingival tooth biofilm was collected using sterile wooden toothpicks, pooled by subject in 100 µl TE-buffer (10 mM Tris, 1 mM EDTA, pH 7.6), and stored at −80 °C until DNA extraction and 16S rDNA amplicon sequencing by Illumina MiSeq and PacBio RS II SMRT (Pacific Biosciences, Menlo Park, CA, USA) was conducted.

### Caries scoring and lifestyle information

The number of teeth, cavitated carious lesions (D), fillings (F), and missing (M) tooth surfaces were scored from visual and radiograph examinations in dental clinics. The sum of the decayed and filled tooth surfaces (DFS) was calculated (caries prevalence). The M-component was not included in the caries index because losses of teeth were due to orthodontic or tooth hypomineralization reasons. The caries incidence (increment) was calculated as the DFS increase from 17 to 19 years of age. Information on the general health status, medication, oral hygiene, dietary habits, and tobacco use (smoking and snuff) was obtained by a questionnaire when the adolescent was 17 years old.

### Mock communities

Three mock communities of oral species were created. These included (*i*) 20 *Lactobacillus* species (*L. acidophilus, L. brevis, L. buchneri, L. casei, L. coleohominis, L. crispatus, L. curvatus, L. fermentum, L. gallinarium, L. gasseri, L. graminis, L. jensenii, L. johnsonii, L. panis, L. paracasei, L. pentosus, L. reuteri, L. rhamnosus, L. salivarius, and L. vaginalis*); (*ii*) 10 *Streptococcus* species, (*S. gordonii, S. intermedius, S. mitis, S. mitis bv 2, S. mutans, S. oralis, S. parasanguinis I, S. salivarius, S. sanguinis, and S. sobrinus*); and (*iii*) 25 species of mixed genera (*Actinomyces gerencseriae, Actinomyces meyeri, Actinomyces odontolyticus, Bifidobacterium longum, Escherichia coli, Fusobacterium nucleatum, L. acidophilus, L. casei, L. coleohominis, Leptotrichia buccalis, Porphyromonas gingivalis, Prevotella denticola, Prevotella oris, Rothia dentocariosa, Scardovia wiggsiae, S. gordoni, S. intermedius, S. mitis, S. mutans, S. oralis, S. parasanguinis I, S. salivarius, S. sanguinis, S. sobrinus, and Veillonella parvula*). Equal aliquots were obtained from each bacterial suspension (OD_600_ = 2.0), resulting in a total volume of 1 ml. All 3 mocks were used for Illumina MiSeq sequencing, and the *Streptococcus* and *Lactobacillus* mocks for PacBio SMRT sequencing.

### DNA extraction

Genomic DNA was extracted from the saliva pellets, tooth biofilm samples, 3 mock communities, and a sample of ultra-pure water (negative control) with a Gene elute™ Bacterial Genomic DNA kit (Sigma-Aldrich, St. Louis, MO, USA) as described previously^11^. Briefly, samples were (*i*) centrifuged for 5 min at 13,000 rpm, (*ii*) lysed in buffer with lysozyme and mutanolysin for 30 min at 37 °C, (*iii*) treated with RNase for 2 min at room temperature followed by Proteinase K for 10 min at 55 °C, (*iv*) mixed with ethanol and transferred to the binding column, and (*v*) washed and eluted in 100 µl of elution buffer. The DNA quality and quantity were evaluated using a Nanodrop 1000 spectrophotometer (Thermo Scientific, Wilmington, DE, USA) to meet the standard set by the sequencing facilities (OD 260/280 ratio ≥1.8). DNA was extracted from undivided samples, and after the DNA content had been determined, the volumes at concentrations requested by the sequencing facilities were subjected to further preparation.

### Sequencing

Multiplex 16S rDNA amplicon sequencing was performed using two different platforms, Illumina MiSeq (http://www.illumina.com) and PacBio RS II SMRT (http://www.pacb.com/smrt-science/smrt-sequencing/). The former analyses were performed at Forsyth Research Institute (Cambridge, MA, USA), and the latter at GATC Biotech AG (Konstanze, Germany).

For Illumina MiSeq sequencing, the HOMINGS protocol was applied as described previously^30^. Briefly, the V3-V4 hypervariable regions of the 16S rDNA were PCR amplified using the forward 341F (AATGATACGGCGACCACCGAGATCTACACTATGGTAATTGT*CCT* **ACGGGAGGCAGCAG**) and reverse 806R (CAAGCAGAAGACGGCATACGAGATNNNNNNNNNNNNAGTCAGTCAGCC**GGACTACHVGGGTWTCTAAT**) primers (primer sequences are in bold, and sample-specific sequence tags (barcodes) are indicated by NNNNNNNNNNNN). Amplicons were purified (AMPure beads; Beckman Coulter Genomics, Danvers, MA); 100 ng of each library was pooled, gel purified, and quantified; and 20% PhiX was added to 12 pmol of the library mixture and run on the MiSeq system. Pair-end reads were fused; barcodes, primers, and ambiguous and chimeric sequences were removed; and taxa were identified by the ProbeSeq customized BLAST programme (HOMINGS, http://homings.forsyth.org/index2.html) for the recognition of 538 species by 638 probes of 17 to 40 bases followed by the detection of 129 genus-level probes for closely related species. Species/phylotypes targeted by the Genus probes are described at http://homings.forsyth.org/index2.html. Taxa represented by <10 sequences were excluded.

In parallel, the QIIME pipeline (version 1.8.0) was applied to raw sequences for comparisons, i.e., sequences were quality filtered according to QIIME default values and binned into clusters (OTUs) using UCLUST at 97.0% similarity. Potential chimaeras were removed (USEARCH), and taxonomic determination was performed by BLAST against the HOMD database for oral bacteria (www.HOMD.org). The named or unnamed species or phylotypes were identified by their HOMD Human Oral Taxon (HOT) identity. OTUs with <10 sequences were excluded.

For PacBio SMRT amplicon sequencing, hairpin adaptors were ligated to V1-V8 hypervariable regions of the 16S rDNA, sample barcodes were added to the end of the amplicons, and standard PacBio SMRT bell adapters were ligated to the barcoded amplicon^24^. The obtained sequences were quality filtered as described above, and potential chimaera sequences were removed. These steps were performed at GATC. Chimaera-free OTU clusters were taxonomically identified by BLAST against the HOMD database at 98.5% similarity.

The original sequencing data are available from the Figshare (accession link: 10.6084/m9.figshare.4552849).

### Complementary PCR and cultivation

The presence of *S. mutans* in saliva and tooth biofilm was evaluated by PCR using the KAPA2G Robust HotStart PCR Ready Mix (2´) kit (Kapa Biosystems, Boston, MA, USA) as described previously^11^. In addition, colony forming units (CFUs) per ml of saliva for mutans streptococci (*S. mutans* and *S. sobrinus*) were assessed in fresh saliva by cultivation on mitis salivarius sucrose agar supplemented with 0.2 U of bacitracin (Becton, Dickinson and Company, Stockholm, Sweden) and incubated at 37 °C in 5% CO_2_ for 48 h.

### Statistical analyses

Normally distributed variables are presented as means with 95% confidence intervals, and group differences were analysed using ANOVA or unpaired *t*-tests. The bacterial taxa mean percentage of all sequences (abundance) and the proportion of adolescents in whom the taxa were identified (prevalence) were calculated and tested with non-parametric tests (Mann-Whitney U and Chi-squared/Fisher’s exact test, respectively). Correlations were evaluated by Spearman correlation coefficients. The mean caries increment was adjusted by sex, BMI, sugar intake, and oral hygiene in general linear modelling (glm). These analyses were performed using SPSS version 23 (IBM Corporation, Armonk, NY, USA).

P-values of taxa prevalence comparisons were evaluated with Benjamini-Hochberg corrections for multiple testing, i.e., p-values ≤ 0.001 were considered significant. For other comparisons, a p-value <0.05 was considered significant. Tests were two-sided.

Rarefaction curves plotting the number of observed species as a function of the number of sequences per sample were established to compare the microbial richness (α diversity) among the samples. Principal component analysis (PCA) was used to explore the clustering of subjects by the ProbeSeq taxa, and principal coordinate analysis (PCoA) was used to define clustering by QIIME identified OTUs.

The Linear Discriminant Analysis Effect Size (LEfSe) algorithm was used to identify taxa (genus level or higher) that differed in relative abundance between the two groups^38^. The online Galaxy Version 1.0 interface (http://huttenhower.sph.harvard.edu) was used, and the threshold for the logarithmic LDA score was set at 1.0. The results are displayed in a cladogram and a bar graph.

Partial least square (PLS) regression was used to identify taxa associated with the caries status. The PLS models included taxa prevalence and potential lifestyle confounders, i.e., sex, BMI, oral hygiene, sugar intake, and tobacco use. The results are presented as PLS correlation coefficients from a column loading plot. The variables for which the 95% CI did not include zero were considered significant. SIMCA P+ version 12.0 (Umetrics AB, Umeå, Sweden) was used for PCA and PLS.

### Ethical approval

The study was approved by the Regional Ethical Review Board in Umeå, Sweden (Dnr 2012-111-31 M) with an addendum for the 2-year follow up (Dnr 2015-389-32 M). All experiments and data collections were performed in accordance with relevant guidelines and regulations, including that all adolescents and their caregivers provided informed consent and data collection and handling followed the Helsinki declaration and the Swedish Law on personal data act (PuL).

## Electronic supplementary material

Supplementary figures S1 and S2

Dataset 1
