# Supplementary material for: Saliva and tooth biofilm bacterial microbiota in adolescents in a low caries community
Source: Sci Rep. 2017 Jul 19;7:5861. doi: 10.1038/s41598-017-06221-z (PMC5517611; doi:10.1038/s41598-017-06221-z)

Supplementary Info File

Saliva and tooth biofilm bacterial microbiota in adolescents in a low caries community

Linda Eriksson[1,2], Pernilla Lif Holgerson[2], Ingegerd Johansson[1*]

[1]Department of Odontology/section of Cariology, Umeå University, Umeå, Sweden

[2]Department of Odontology/section of Pedodontics, Umeå University, Umeå, Sweden

*To whom correspondence should be addressed: I. Johansson, Department of Odontology, Umeå University, Umeå, Sweden
Mail: Department of Odontology, Umeå University, SE 90187 Umeå, Sweden
E-Mail: ingegerd.johansson@umu.se

Supplementary Figure S1. PCoA plot for QIIME identified OTUs in saliva extracted DNA.

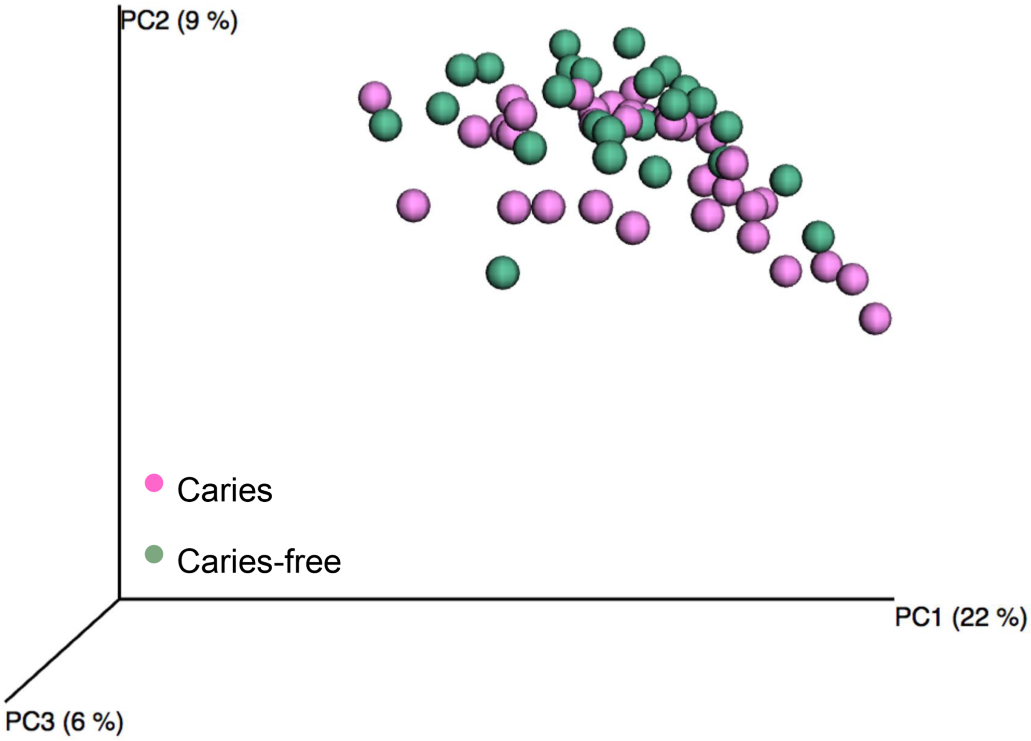

Supplementary Figure S2. Receiver operating characteristic (ROC) curve with the true positive rate (sensitivity) against the false positive rate (specificity) for *S. mutans* detection by Illumina MiSeq versus PCR in saliva is plotted. The sensitivity for saliva and tooth biofilm were 0.98 and 0.90, respectively. The area under the curve was 0.94 for saliva and 0,74 for tooth biofilm.

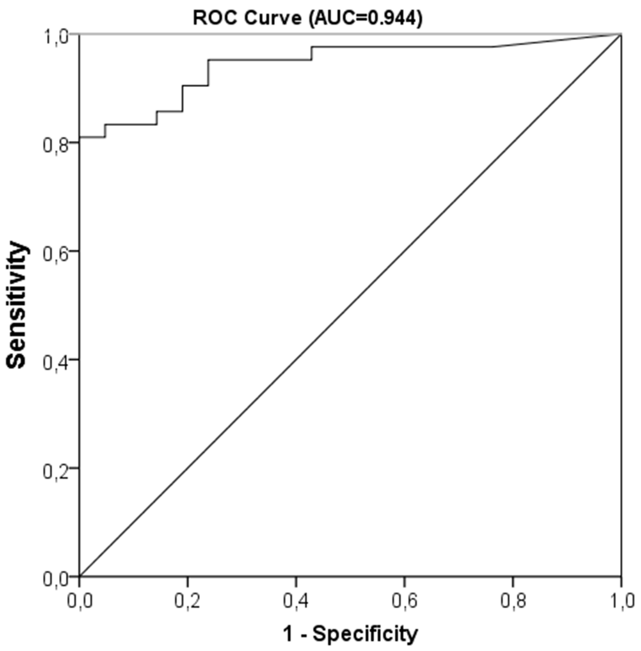

Supplement: Supplementary file 1 — Supplementary figures S1 and S2 [file 41598_2017_6221_MOESM1_ESM.pdf]
